# Investigation of the impact of planar microelectrodes on macrophage-mediated mesenchymal stem cell osteogenesis

**DOI:** 10.3389/fcell.2024.1401917

**Published:** 2024-06-03

**Authors:** Hui Chen, Chuchu Xu, Qin Huang, Yuhua Chen, Kui Cheng, Huiming Wang, Xiaoyi Chen

**Affiliations:** ^1^ School of Medicine, Zhejiang University, Hangzhou, China; ^2^ Stomatology Hospital, School of Stomatology, Zhejiang University School of Medicine, Zhejiang Provincial Clinical Research Center for Oral Diseases, Key Laboratory of Oral Biomedical Research of Zhejiang Province, Cancer Center of Zhejiang University, Engineering Research Center of Oral Biomaterials and Devices of Zhejiang Province, Hangzhou, China; ^3^ School of Materials Science and Engineering, State Key Laboratory of Silicon Materials, Zhejiang University, Hangzhou, China

**Keywords:** planar microelectrode, surface charge, macrophage, mesenchymal stem cells, polarization phenotype, osteogenesis

## Abstract

Osseointegration commences with foreign body inflammation upon implant placement, where macrophages play a crucial role in the immune response. Subsequently, during the intermediate and late stages of osseointegration, mesenchymal stem cells (MSCs) migrate and initiate their osteogenic functions, while macrophages support MSCs in osteogenesis. The utilization of ferroelectric P(VDF-TrFE) covered ITO planar microelectrodes facilitated the simulation of various surface charge to investigate their effects on MSCs’ osteogenic differentiation and macrophage polarization and the results indicated a parabolic increase in the promotional effect of both with the rise in piezoelectric coefficient. Furthermore, the surface charge with a piezoelectric coefficient of −18 exhibited the strongest influence on the promotion of M1 polarization of macrophages and the promotion of MSCs’ osteogenic differentiation. The impact of macrophage polarization and MSC osteogenesis following the interaction of macrophages affected by surface charge and MSC was ultimately investigated. It was observed that macrophages affected by the surface charge of −18 piezoelectric coefficient still exerted the most profound induced osteogenic effect, validating the essential role of M1-type macrophages in the osteogenic differentiation of MSCs.

## 1 Introduction

Oral implant technology has emerged as a significant approach for tooth restoration owing to its minimal damage to adjacent teeth and high long-term success rates. The achievement of good osseointegration of dental implants within the alveolar bone is pivotal for the success of implant surgery.

Brånemark et al. (1969) defines osseointegration as the direct attachment of the implant to the newly formed and well-developed bone tissue nearby, without any intervening fibrous tissue at the interface. Mounting evidence (Li et al., 2018; Albrektsson et al., 2019) indicates that osseointegration involves immune-regulated bone tissue regeneration, rather than just bone remodeling. The initiation of osseointegration is characterized by an inflammatory response, in which various immune cells participate. Macrophages (Mφ) play a vital role in this process by polarizing into different phenotypes and secreting various signaling molecules that can induce differentiation of mesenchymal stem cells (MSCs) into osteoblasts to promote bone formation, or transform into osteoclasts to promote bone resorption (Albrektsson et al., 2018). Following the gradual subsiding of acute inflammation, a multitude of MSCs migrate to the implant surface and differentiate into osteoblasts under the influence of intracellular and extracellular bioactive factors (Bianco and Robey, 2015). Throughout the process of osseointegration, the interaction between macrophages and MSCs, as well as their appropriate functioning, holds great importance for successful osseointegration (Salvi et al., 2015).

The surface potential, including surface charge and surface electric field, of electroactive materials can regulate cell adhesion, growth, migration, and functioning by mimicking endogenous electrical signaling molecules or electrical pulse signals (Hench and Polak, 2002). Indium tin oxide (In_2_O_3_-SnO_2_, ITO) (Elfallal et al., 1991), also known as tin-doped indium oxide, is classified as an n-type semiconductor. The fabrication of ITO planar microelectrodes involves depositing ITO on soda lime or silicon boron-based glass substrates using magnetron sputtering, followed by precise design and etching of the ITO surface using laser ablation. Tandon et al. (2010) have innovated a new micro-patterned ITO substrate for fork-finger electrode arrays, allowing for the construction of multiple pairs of electrodes to facilitate the external electrical stimulation of cultured cells. Polyvinylidene fluoride (PVDF) is recognized as a biocompatible material with exceptional mechanical properties and remarkable electroactivity, including pyroelectric, ferroelectric, and piezoelectric properties (Kao et al., 2017). To enhance its ferroelectric and piezoelectric attributes, researchers (Nunes-Pereira et al., 2015) introduced the trifluoroethylene monomer (TrFE) into PVDF, resulting in the copolymer Poly(vinylidene fluoride-trifluoroethylene) (P(VDF-TrFE)), which exhibit improved charge accumulation and storage capabilities, enabling the acquisition of a permanent surface potential.

P(VDF-TrFE) is capable of carrying surface charge and has been demonstrated to promote osteogenesis in MSCs *in vitro* (Luo et al., 2022; Zhang et al., 2022), enhance bone repair *in vivo* (Gimenes et al., 2019; Zhang et al., 2022), and facilitate macrophage polarization (Dai et al., 2021). The application of an external power source through ITO planar microelectrodes can impart a continuous electrical stimulation to cells or tissues, thereby influencing their morphology and function. The P(VDF-TrFE)/ITO planar microelectrodes fabricated by coating P(VDF-TrFE) onto ITO planar microelectrodes can modulate the morphology and function of cells and tissues both *in vitro* and *in vivo* through the adjustment of surface charge polarity and quantity. Moreover, by varying the duration and intensity of the applied voltage, its possible to synergistically or antagonistically modulate the effect of surface charge at specific times. Thus this electroactive material can achieve multidirectional control of surface potential and exert multifaceted effects on cells which makes it a material with significant research potential.

In view of the regulatory effects of surface charge on both macrophages and MSCs, the research aims to investigate the impact of modulating the surface charge of the P(VDF-TrFE)/ITO planar microelectrodes on macrophage polarization, osteogenic differentiation of MSCs, and the interaction between MSCs and macrophages which influenced by the surface charge. The primary objective of the study is to explore targeted regulation of the interaction between macrophages and mesenchymal stem cells to facilitate bone regeneration.

## 2 Materials and methods

### 2.1 Materials

ITO conductive glass was fabricated and processed by South China Xiangcheng Technology Co., Ltd. P(VDF-TrFE) was purchased from Piezotech. N,N-Dimethylformamide (DMF), anhydrous ethanol, chloroform, isopropanol, and methanol were provided by Sinopharm Chemical Reagent Co., Ltd.

### 2.2 Preparation and surface charge construction of P(VDF-TrFE)/ITO planar microelectrodes

A 7% wt P(VDF-TrFE)/DMF solution was prepared and dropped onto the center of the ITO planar microelectrode substrates. The dried P(VDF-TrFE) was then annealed in a muffle furnace to obtain μ-P(VDF-TrFE)/ITO planar microelectrodes (Supplementary Figure S1A). The surface charge was constructed using direct contact polarization (Supplementary Figure S1B), and the piezoelectric constant (d_33_) of the polarized P(VDF-TrFE)/ITO planar microelectrodes was measured using a ZJ-3 piezoelectric tester. The applied voltage strength and the piezoelectric coefficient of the surface of the final constructed material are shown in Table 1.

**TABLE 1:** Surface charge grouping.

Group	Applied voltage intensity (V)	Piezoelectric constant (d_33_,pC/N)
d_33_ = 0 ± 0.5 pC/N	0	0 ± 0.5
d_33_ = −6 ± 0.5 pC/N	3.3	−6 ± 0.5
d_33_ = −12 ± 0.5 pC/N	4	−12 ± 0.5
d_33_ = −18 ± 0.5 pC/N	5.5	−18 ± 0.5
d_33_ = −22 ± 0.5 pC/N	7.5	−22 ± 0.5

### 2.3 Fourier transform infrared spectrometer (FTIR)

FTIR (Vertex 70v) with a wavelength scanning range of 400–4,000 cm^−1^ was used to determine the crystalline properties of P(VDF-TrFE). The β-phase content of P(VDF-TrFE) was calculated using the characteristic absorption bands and coefficients of the α-phase and β-phase according to the following equation:

Fβ=AβKβKαAα+Aβ×100%

F(β):β-phase content, A_α_:absorbance at 766 cm^−1^, A_β_:absorbance at 840 cm^-1^, K_α_:absorption coefficients corresponding to the A_α_ absorbance, with values of 6.1 × 10^4^ cm^2^mol^−1^, K_β_:absorption coefficients corresponding to the A_β_ absorbance, with values of 7.7 × 10^4^ cm^2^mol^−1^ (Kim et al., 1989).

### 2.4 Cell extraction and culture

To achieve a more stable and highly differentiated mesenchymal stem cells (MSCs), we extracted the cells from the bone marrow of 5-week-old male C57BL/6 mice. After complete stripping of the femur and tibia, the mid-portion of the bone marrow was flushed out using complete medium for culture, and the medium was exchanged at a frequency of 2–3 days until the high purity of the MSCs were obtained. MSCs were cultured in α-MEM medium containing 15% fetal bovine serum (FBS, Gibco) and 1% Penicillin-Streptomycin Liquid, and the mouse bone marrow MSCs osteogenic induction differentiation kit (OriCell) was used in the osteogenic differentiation induction process. RAW264.7 macrophages (CTCC-001-0048) were cultured in high-glucose DMEM medium containing 10% FBS. Both cell types were cultured at 37°C in a 5% CO_2_ cell culture incubator.

### 2.5 Cell counting kit-8 (CCK-8) assay

Cell viability was assessed using the CCK-8 assay for both MSCs cultured on the 1st, 4th, 7th and 14th days and RAW264.7 macrophages cultured on 12th hours, 1st, 2nd, 4th and 7th days. The optical density (OD) at 450 nm was measured using an enzyme marker to assess cell viability.

### 2.6 Field emission scanning electron microscopy (FE-SEM)

FE-SEM (Nova Nano 450) was used to observe the morphology of etched ITO electrodes, P(VDF-TrFE)/ITO planar microelectrodes and macrophages grown on the surface of planar microelectrodes carrying different surface charge. Cells needed to be fixed by 2.5% glutaraldehyde solution and 1% osmium acid, dehydrated by ethanol gradient (30%, 50%, 70%, 80%, 90%, 95%, 100%) and dried at zero boundary point. All samples required gold spray coating before observation.

### 2.7 Quantitative real-time polymerase chain reaction (qRT-PCR) assay

The gene expression was measured via qRT-PCR assay. Total RNA was extracted using traditional experimental method, briefly cells were lysed using Trizal, extracted with chloroform, precipitated with isopropanol, washed with 75% ethanol and lysed in DEPC water. Its purity and concentration were tested before reverse transcription and *in vitro* amplification. The cDNA was reverse transcribed by PrimeScript RT Reagent Kit with gDNA Eraser (TaKaRa) and gene expression level detection was performed by MonAmp SYBR Green qPCR Mix (MonAmp). The primers for the target genes were listed in Table 2.

**TABLE 2:** Primers for target genes.

Gene	Forward primer sequence (5′-3′)	Reverse primer sequence (3′-5′)
*Gapdh*	ACT​CTT​CCA​CCT​TCG​ATG​CC	TGG​GAT​AGG​GCC​TCT​CTT​GC
*Cd80*	GAA​GAC​CCT​CCT​GAT​AGC​AAG​AA	GGA​AGA​CGG​TCT​GTT​CAG​CTA​AT
*Ccr7*	GTG​GTG​GCT​CTC​CTT​GTC​ATT​T	CCC​ACG​AAG​CAG​ATG​ACA​GAA​TA
*Il6*	AAC​CGC​TAT​GAA​GTT​CCT​CTC​TG	TGG​TAT​CCT​CTG​TGA​AGT​CTC​CT
*Tnf*	GCC​TCC​CTC​TCA​TCA​GTT​CTA​TG	ACC​TGG​GAG​TAG​ACA​AGG​TAC​AA
*Mrc1*	GTC​AGA​ACA​GAC​TGC​GTG​GA	AGG​GAT​CGC​CTG​TTT​TCC​AG
*Cd163*	TGC​TGT​CAC​TAA​CGC​TCC​TG	TCA​TTC​ATG​CTC​CAG​CCG​TT
*Il10*	GCA​TGG​CCC​AGA​AAT​CAA​GG	TTC​ATG​GCC​TTG​TAG​ACA​CC
*Tgfb1*	CAT​CCA​TGA​CAT​GAA​CCG​GC	GAA​GTT​GGC​ATG​GTA​GCC​CT
*Alpl*	CAT​GAC​ATC​CCA​GAA​AGA​CAC​C	CCT​GGT​AGT​TGT​TGT​GAG​CGT​AAT
*Col1a1*	GAG​AGG​TGA​ACA​AGG​TCC​CG	AAA​CCT​CTC​TCG​CCT​CTT​GC
*Bglap*	GGA​GGG​CAA​TAA​GGT​AGT​GAA​CAG	ATA​GCT​CGT​CAC​AAG​CAG​GGT
*Runx2*	AGC​GGA​CGA​GGC​AAG​AGT​TT	AGG​CGG​GAC​ACC​TAC​TCT​CAT​A
*Sp7*	TCT​GCG​GCA​AGA​GGT​TCA​CT	GCT​GAT​GTT​TGC​TCA​AGT​GGT​C

### 2.8 Protein activity assay

RAW264.7 macrophage protein expression was detected using Western blot assay, and MSCs protein activity was detected using Alkaline Phosphatase (ALP) quantitative assay kit and Osteocalcin (OCN) enzyme-linked immunosorbent assay (ELISA) kit following the manufacturer’s instructions. Western blot experiments are completed by protein sample collection, concentration determination and normalization, electrophoresis, membrane transfer, containment, primary and secondary antibody incubation, and finally exposure and strips analysis.

### 2.9 Alizarin red staining

The mineralization of precursor osteoblasts was observed using the alizarin red staining method after MSCs were cultured for 14 days. MSCs were first fixed with 4% paraformaldehyde, then stained with alizarin red after washing and finally observed under the microscope when the excess dye was washed off.

### 2.10 Statistical analysis

Data were analyzed using the SPSS 27.0 (SPSS27.0, IBM, USA). Comparisons between inter groups were performed using one-way analysis of variance (ANOVA) or two-way ANOVA and the *post hoc* multiple comparisons were conducted through least significant difference (LSD) when normally distributed or Tamhane’s T2 test when non-normally distributed. *p* values of <0.05 were considered statistically significant and *p* values of <0.01 were considered statistically highly significant.

## 3 Results

### 3.1 Characterization of P(VDF-TrFE)/ITO planar microelectrodes

**FIGURE 1:**
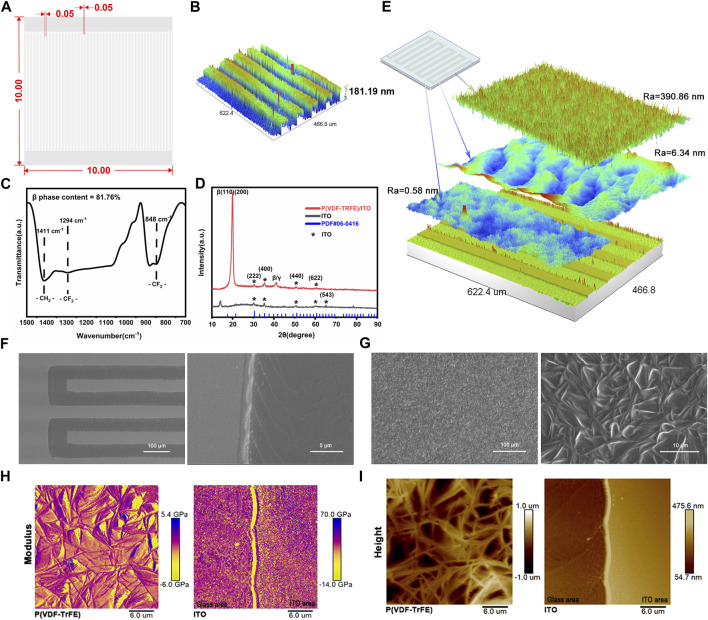
Physicochemical properties of ITO and P(VDF-TrFE) films. **(A)** Schematic diagram of fork finger electrode (10 mm × 10 mm) with width and spacing of 50 µm. **(B)** 3D simulation of fork finger electrode. **(C)** FTIR pattern of P(VDF-TrFE) thin film. **(D)** X-ray Diffraction (XRD) pattern of P(VDF-TrFE)/ITO and ITO planar microelectrode. **(E)** 3D simulation of P(VDF-TrFE)/ITO planar microelectrode. Ra means surface roughness. **(F)** Scanning electron microscopy (SEM) of ITO planar microelectrode (200× and 5,000×). **(G)** SEM of P(VDF-TrFE) film (500× and 3,000×). **(H)** The elastic modulus of ITO planar microelectrodes and P(VDF-TrFE) thin film. **(I)** The surface topography of ITO planar microelectrodes and P(VDF-TrFE) thin film through Atomic Force Microscopy (AFM). N = 3.

The ITO conductive glass underwent laser etching on the surface to create 50 pairs of fork-finger electrodes (Figures 1A, B). The average height disparity between the electrode area (ITO area) and the etched area (glass area) was measured in 181.19 nm and the thickness of P(VDF-TrFE) film was 24.58 μm (Supplementary Figure S2A). The FTIR pattern of P(VDF-TrFE) films reveals three distinct β-phase absorption peaks (Figure 1C), indicating a remarkably high β-phase content of 81.76%. In the microelectrode X-ray Diffraction (XRD, Shimadzu-6000) patterns (Figure 1D), multiple characteristic peaks (*) of ITO are evident, alongside a significant characteristic peak of β-PVDF on the P(VDF-TrFE)/ITO planar microelectrode (2θ = 20°), confirming the high degree of crystallinity and strong piezoelectric properties of the P(VDF-TrFE) films. Utilizing 3D interference microscopy (Veeco, NT9100), a simulation of P(VDF-TrFE)/ITO planar microelectrodes was conducted (Figure 1E), revealing a homogenous surface of P(VDF-TrFE) with a roughness of approximately 390.86 nm. The electrode area demonstrated a cluster-like distribution of particles, while the etched area displayed regular undulations; the roughness of both areas differed but was considerably lower than that of the P(VDF-TrFE). Scanning electron microscopy (SEM) observation unveiled the granular nature of the ITO in the electrode area and pronounced cascading etching traces in the glass area, with a distinct and precise boundary between the two regions (Figure 1F). Furthermore, the P(VDF-TrFE) film displayed more uniform needle-like whiskers (Figure 1G). The surface morphology observed by Atomic Force Microscopy (AFM) displayed similarities to that observed by SEM, and a large number of pores can also be observed on the surface of the thin film (Figure 1I). The elasticity modulus of the P(VDF-TrFE)/ITO planar microelectrodes was measured using an AFM probe to characterize the surface stiffness. The elasticity modulus within the ITO areas of the ITO planar microelectrodes was similar to those in glass areas, and the whole had an average value of 54.14 GPa, significantly higher than that of the P(VDF-TrFE) films whose average elasticity modulus was 5.58 GPa (Figure 1H).

### 3.2 The impact of surface charge on the osteogenic induction of MSCs

**FIGURE 2:**
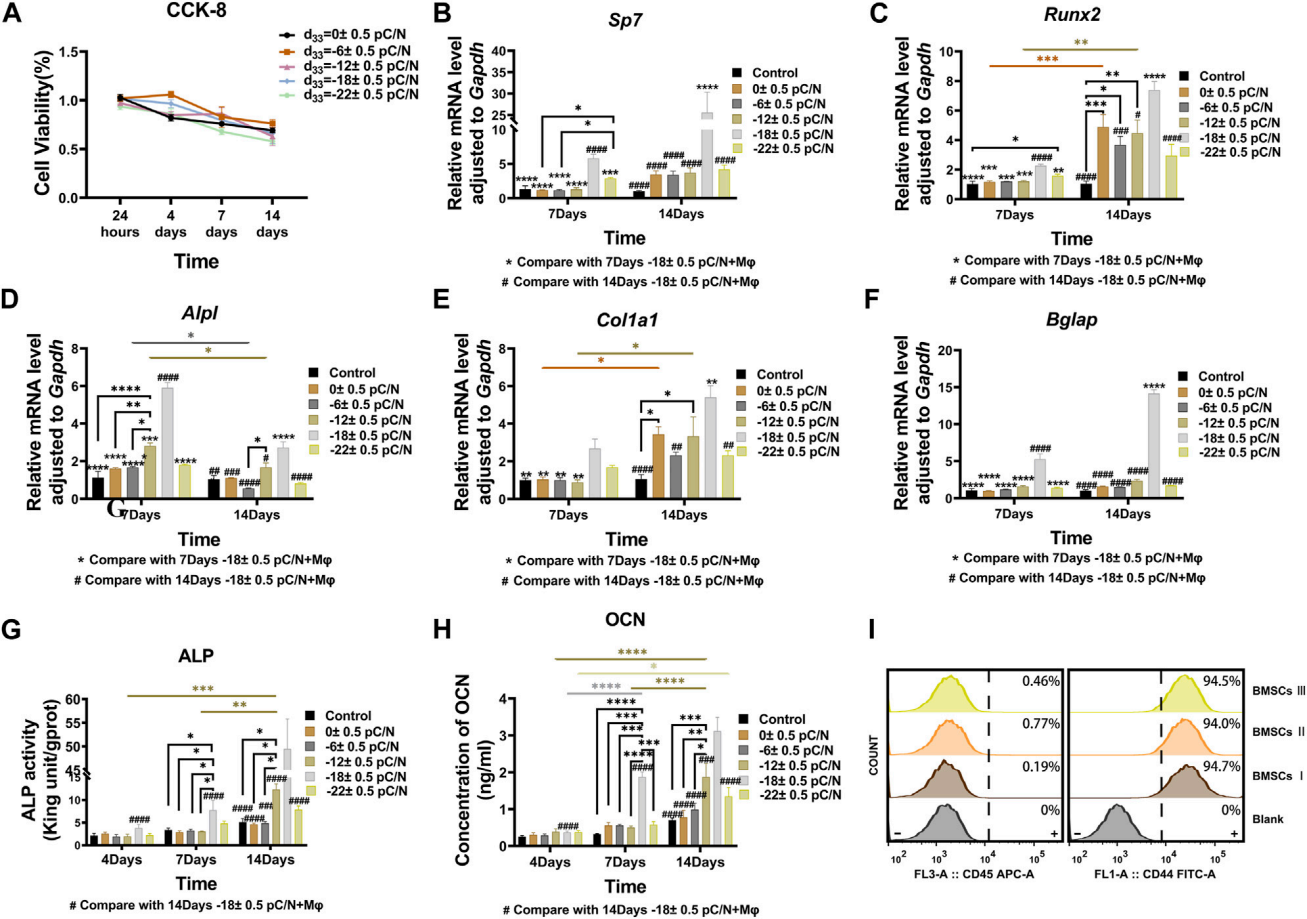
MSCs were cultured on P(VDF-TrFE)/ITO planar microelectrodes with varying surface charge to investigate surface charge effects on the osteogenic induction of MSCs. **(A)** Cell viability was examined after 1, 4, 7, and 14 days of culture using the CCK-8 assay, with the cell viability of the Control group serving as reference. **(B–F)**The gene expressions of *Sp7*, *Runx2*, *Alpl*, *Col1a1* and *Bglap* were evaluated by qRT-PCR at 7 and 14 days of culture. **(G)** The ALP activity was differentially analyzed at 4, 7, and 14 days of culture. **(H)** The concentration of OCN in the cell culture supernatant was differentially analyzed at 4, 7, and 14 days, with the supernatant collected each time to ensure that OCN accumulated for 2 days. **(I)** Purity of extracted primary BMSCs was tested using flow cytometry by CD45 and CD44 surface protein. The Control group comprised cells cultured without material. The results are presented as mean ± SD and comparised with two-way ANOVA and *post hoc* multiple comparisons through LSD or Tamhane’s T2 test, with *p < 0.05, **p < 0.01, ***p < 0.001, ****p < 0.0001 denoting significance levels, n = 3.

**FIGURE 3:**
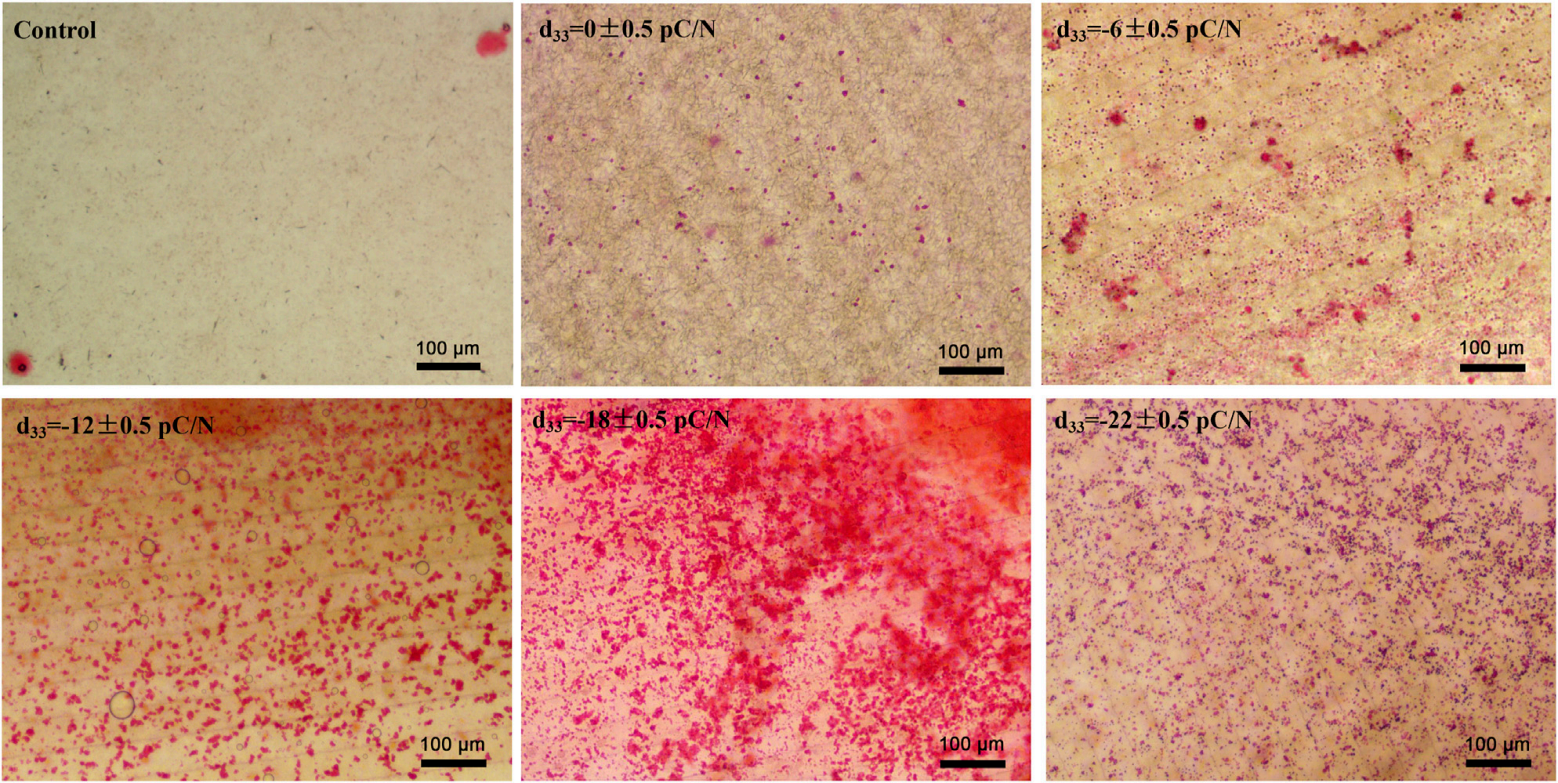
Extracellular matrix mineralization was observed after culturing MSCs on P(VDF-TrFE)/ITO planar microelectrodes with varying surface charge for 14 days. Cells cultured without material were Control group, n = 3.

MSCs were cultured on P(VDF-TrFE)/ITO planar microelectrodes with varying surface charge. The cell viability was evaluated on days 1, 4, 7, and 14 of the culture period. No significant differences in cell activity were observed between the different groups after 24 h of adhesion. However, as the culture time progressed, a noticeable and gradual decrease in cell viability was evident across all groups (Figure 2A). Flow cytometry was used to detect the purity of primary BMSCs extracted from mice after three times cell passaging and showed that CD45 marked cells could barely find and nearly all cells could targeted by CD44 marker (Figure 2I). Alkaline phosphatase (ALP), collagen type I (COL1) and osteocalcin (OCN) are osteogenesis-specific matrix proteins that can reflect the activity of osteoblasts and the function of bone formation. ALP is highly expressed at the early stage of osteoblast differentiation and maturation. COL1 which is a mid-stage marker is highly expressed in the osteogenesis stage. OCN which belongs to the late stage markers appears at the end of osteoblast differentiation and can bind to Ca^2+^ to regulate calcium homeostasis and bone mineralisation. Osteogenesis-related transcription factors osterix (*Sp7*) and runt-related transcription factor 2 (*Runx2*) are essential for regulating the expression of osteogenesis-specific matrix protein genes, and are full term markers of osteogenesis. Evaluation of the expression of osteogenesis-related genes, including *Sp7*, *Runx2*, *Alpl*, *Col1a1* and *Bglap* at 7 and 14 days of culture, revealed that negative surface charge could enhance the expression of osteogenic genes in MSCs, with the d_33_ = −18 ± 0.5 pC/N group displaying significantly higher expression than the other groups (Figures 2B–F). Variations in *Alpl* gene expression (Figure 2D) were predominantly observed on the 7th day, while differences in *Col1a1* and *Runx2* gene expression (Figures 2C, E) were more prominent on the 14th day. Conversely, differences in *Bglap* and *Sp7* gene expression (Figures 2B, F) were insignificant in all groups except for the d_33_ = −18 ± 0.5 pC/N group. At the protein level, the osteogenic induction effect of surface charge on MSCs was assessed by measuring the concentration of ALP in the cells and OCN in the cell culture supernatant on days 4, 7, and 14 of culture (Figures 2G, H). Notably, only the d_33_ = −18 ± 0.5 pC/N group exhibited markedly high activity of ALP and OCN on the 7th day, with significant differences in protein expression among all groups appearing on the 14th day, reaching maximum levels at a surface charge of d_33_ = −18 ± 0.5 pC/N. Furthermore, after 14 days of incubation, the extracellular matrix was stained with alizarin red (Figure 3). In the Control group, only a few isolated calcium nodules were observed under the microscope. However, as the quantity of negative surface charge on the planar microelectrode increased, the number of calcium nodules on the surface gradually escalated. The d_33_ = −18 ± 0.5 pC/N group displayed numerous connected calcium nodules, while the d_33_ = −22 ± 0.5 pC/N group exhibited numerous but disconnected calcium nodules.

### 3.3 The impact of surface charge on macrophage polarization

**FIGURE 4:**
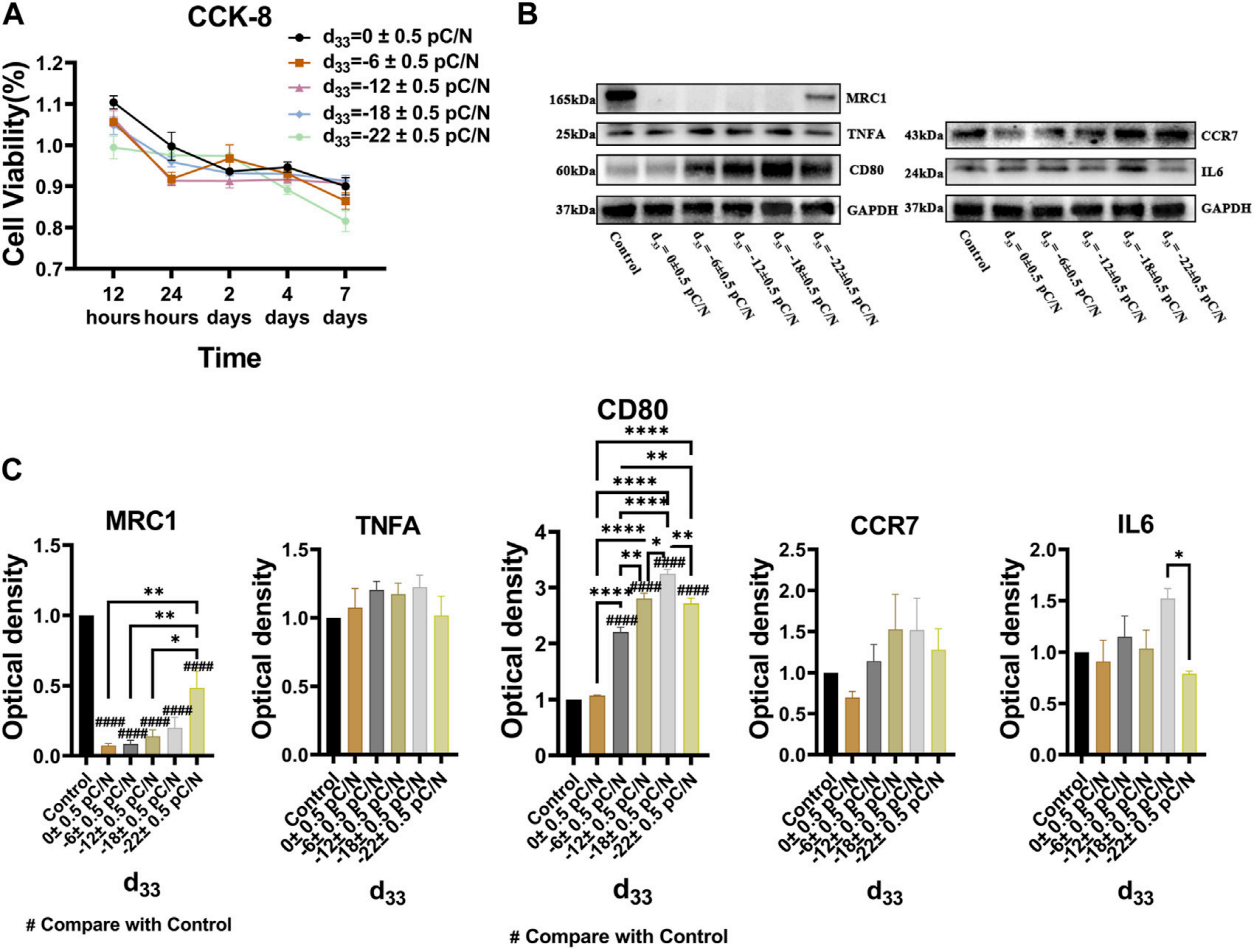
The cell viability and protein influence of surface charge on the polarization of RAW264.7 macrophages was investigated by cultivating them on P(VDF-TrFE)/ITO planar microelectrodes with varying surface charge. **(A)** The cell viability was assessed by the CCK-8 assay after 12 h, 1, 2, 4, and 7 days of culture, with the cell viability of the Control group serving as a reference. **(B)** The level of MRC1, TNFA, CD80, CCR7 and IL6 proteins was analyzed by Western blot after 4 days of culture and the quantification of Western blot results were shown in **(C)**. The Control group comprised cells cultured without material. The results are presented as mean ± SD and comparised with one-way ANOVA and *post hoc* multiple comparisons through LSD or Tamhane’s T2 test, with *p < 0.05, **p < 0.01, ***p < 0.001, ****p < 0.0001 denoting significance levels, n = 3.

**FIGURE 5:**
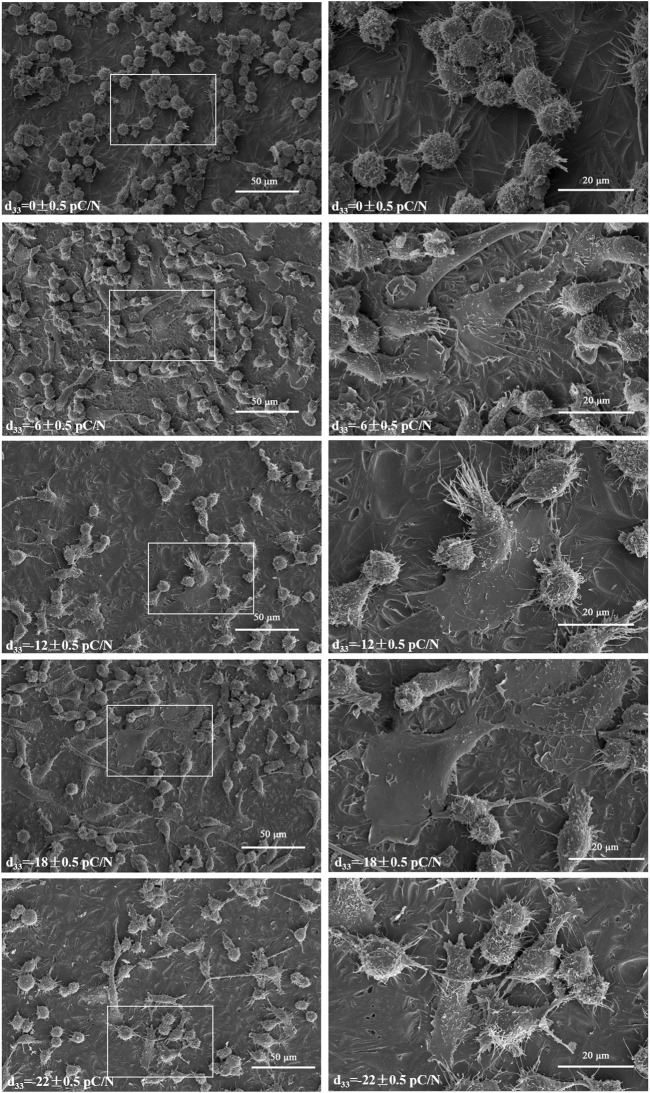
Initial observations of RAW264.7 macrophages were made using SEM after 1 day of culture on P(VDF-TrFE)/ITO planar microelectrodes with varying surface charge (500× and 1,500×), n = 3.

**FIGURE 6:**
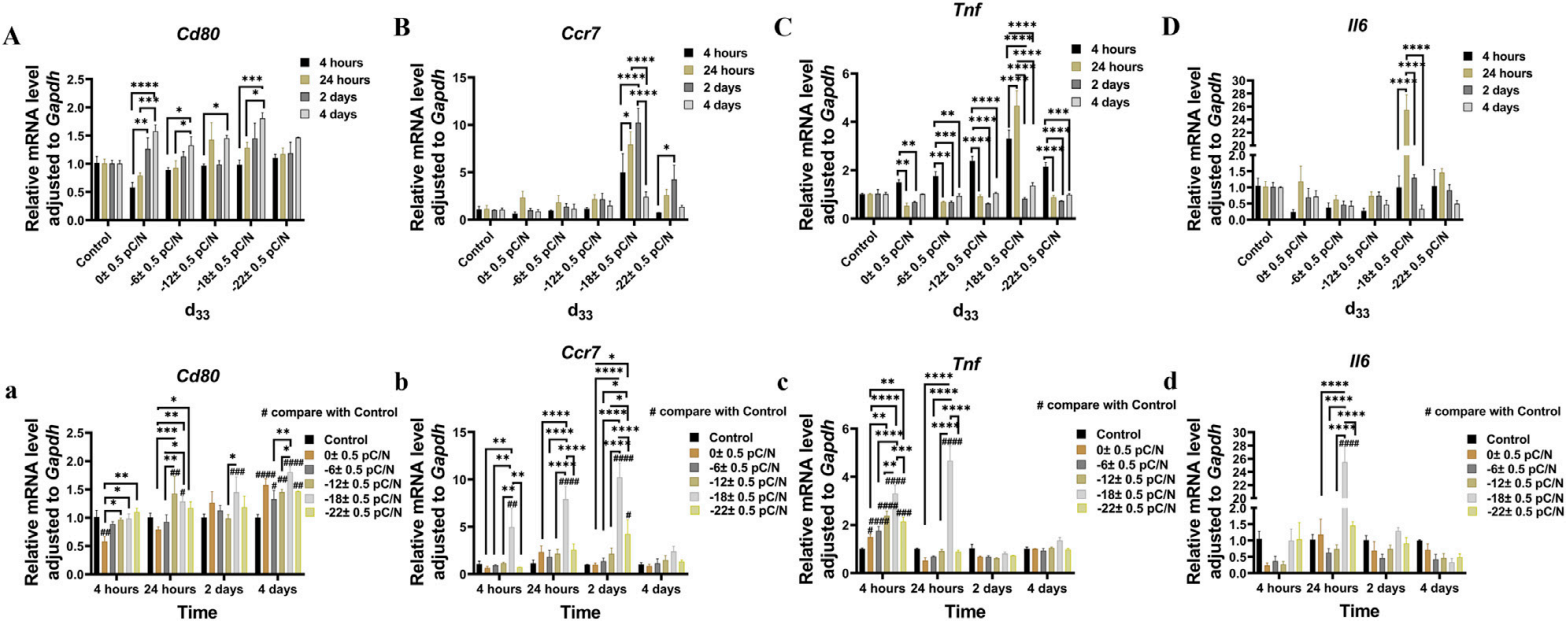
The RAW264.7 macrophages gene expression of *Cd80* **(A,a)**, *Ccr7* **(B,b)**, *Tnf* **(C,c)** and *Il6* **(D,d)** was studied using qRT-PCR at 24 h, 1, 2, and 4 days of culture on P(VDF-TrFE)/ITO planar microelectrodes with varying surface charge. The Control group comprised cells cultured without material. The results are presented as mean ± SD and comparised with one-way ANOVA and *post hoc* multiple comparisons through LSD or Tamhane’s T2 test, with *p < 0.05, **p < 0.01, ***p < 0.001, ****p < 0.0001 denoting significance levels, n = 3.

RAW264.7 macrophages cultured on P(VDF-TrFE)/ITO planar microelectrodes with various surface charge. RAW264.7 macrophages were assessed for cell viability at 12 h, 1, 2, and 4 days of culture and exhibited a transient pro-proliferative effect in all groups except the d_33_ = −22 ± 0.5 pC/N group, which directly suppressed RAW264.7 growth at 12 h. Following 24 h, a slight inhibition of cell proliferation was observed, with a notably decreasing trend in the d_33_ = −22 ± 0.5 pC/N group (Figure 4A). SEM examination after 1 day of culture revealed that the surface of uncharged material (d_33_ = 0 ± 0.5 pC/N group) largely comprised unpolarized small spherical M0 macrophages. As the surface charge increased, an elevated number of irregularly flattened, polarized macrophages emerged. The “fried egg"-like or “polygonal star"-like macrophages represented the M1-type, while the “long shuttle"-type macrophages denoted the M2-type. The d_33_ = −18 ± 0.5 pC/N group was dominated by the polarisation of macrophages of M1-type and the d_33_ = −22 ± 0.5 pC/N group was dominated by macrophages of M2-type (Figure 5). After 4 days of cultivation, protein activity including MRC1, CD80, CCR7, TNFA and IL6 was evaluated (Figures 4B, C). The surface marker MRC1 of M2 macrophages exhibited strong expression in the Control group, limited expression in the d_33_ = −22 ± 0.5 pC/N group, and virtually no expression in the other groups. The expression of the M1-type macrophage surface markers CD80 and CCR7 displayed a parabolic trend with increasing surface charge, with the d_33_ = −18 ± 0.5 pC/N group reaching maximum values. The pro-inflammatory cytokine IL6 exhibited significant expression in the d_33_ = −18 ± 0.5 pC/N group. Dynamic changes in macrophage gene expression of *Cd80*, *Ccr7*, *Tnf* and *Il6* were observed over 24 h, 1, 2, and 4 days of culture (Figure 6), with an increased expression of *Cd80* over time (Figure 6A). High expression of *Il6* and *Tnf* was primarily observed in the initial 2 days of culture (Figures 6C, D). All M1-type macrophage markers were notably expressed at a surface charge of d_33_ = −18 ± 0.5 pC/N (Figures 6B, A–D).

### 3.4 Influences of indirect co-culture of macrophages under diverse surface charge and MSCs on macrophage polarization and osteogenic differentiation of MSCs

**FIGURE 7:**
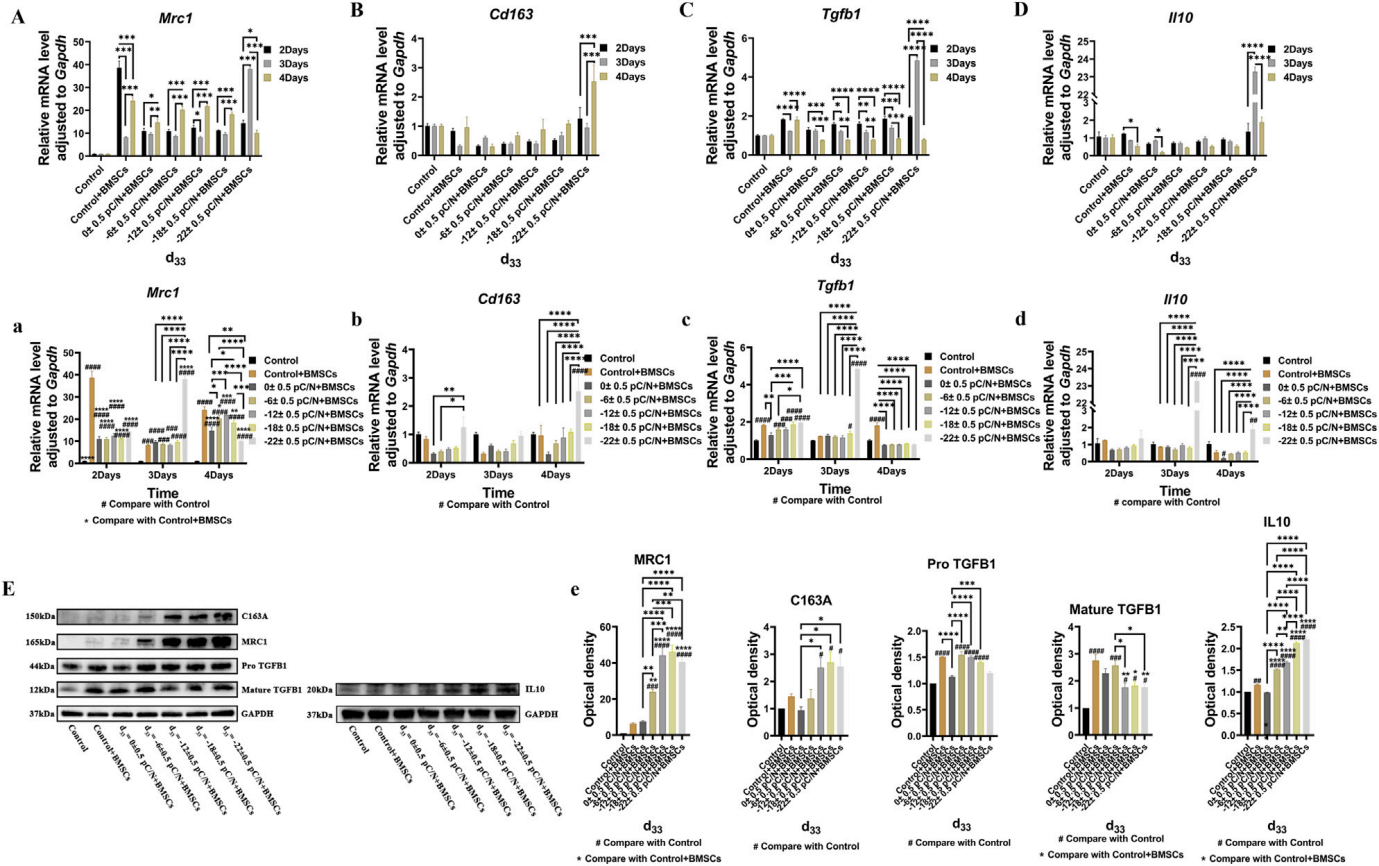
Macrophage polarization effects were evaluated through the indirect co-culture with RAW264.7 macrophages and BMSCs in which macrophages influenced by P(VDF-TrFE)/ITO planar microelectrodes with varying surface charge. **(A–D, a–d)** The expression of *Mrc1*, *Cd163*, *Tgfb1*, and *Il10* in RAW264.7 macrophages were assessed through qRT-PCR on the 2nd, 3rd, and 4th days of co-culture. **(E)** MRC1, C163A, TGFB1, and IL10 protein level in RAW264.7 macrophages were assessed using Western blot after 4 days of co-culture and the quantification of Western blot results were shown in **(e)**. RAW264.7 macrophages were cultured alone without using materials as Control group, and co-cultured without using materials as Control + BMSCs group. The results are presented as mean ± SD and comparised with one-way ANOVA and *post hoc* multiple comparisons through LSD or Tamhane’s T2 test, with *p < 0.05, **p < 0.01, ***p < 0.001, ****p < 0.0001 denoting significance levels, n = 3.

Using a small chamber with a pore size of 0.4 μm (Labselect, China) to culture cells, the upper chamber was seeded with MSCs, while the lower chamber housed RAW264.7 macrophages on P(VDF-TrFE)/ITO planar microelectrodes with varying surface charge. The proportion of MSCs and RAW264.7 macrophages was 1:5 (Lu et al., 2017). In this co-culture process, *Mrc1* gene expression was significantly elevated in the Control + BMSCs group on the second day but notably reduced on the third day, whereas the d_33_ = −22 ± 0.5 pC/N + BMSCs group demonstrated a significant increase on the third day (Figure 7A, a). The early gene expression of anti-inflammatory cytokines *Tgfb1* and *Il10* was more active (Figures 7C, D). The d_33_ = −22 ± 0.5 pC/N + BMSCs group consistently exhibited the strongest M2 phenotypic polarization and the Control group always had the least M2 phenotypic macrophages, while no significant difference in M2 gene expression was observed between the other groups (Figures 7A, a, B, b, C, c, D, d). Additionally, after 4 days of co-culture, the protein expressions of MRC1 and C163A, both were M2 macrophage surface markers, as well as the anti-inflammatory cytokine IL10, increased with the magnitude of surface charge. The Western blot results of TGFB1 exhibited a double band divided into pro-TGFB1 and mature-TGFB1, and the Control group secreted significantly less TGFB1 compared to the other groups (Figures 7E, e).

**FIGURE 8:**
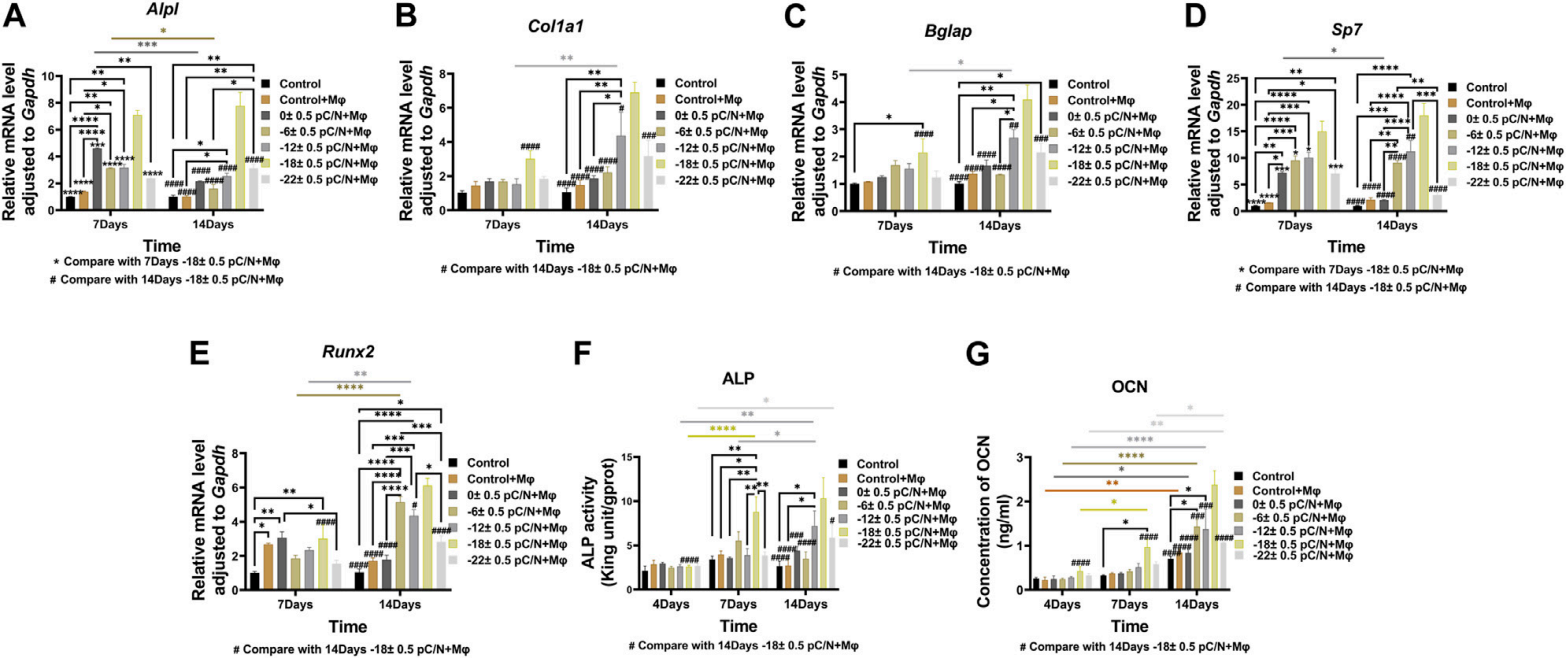
Osteogenic differentiation of MSCs were evaluated through the indirect co-culture with RAW264.7 macrophages and BMSCs in which macrophages influenced by P(VDF-TrFE)/ITO planar microelectrodes with varying surface charge. **(A–E)** The gene expressions of *Alpl*, *Col1a1*, *Bglap*, *Sp7* and *Runx2* were detected by qRT-PCR at 7 and 14 days of co-culture. **(F)** Differential analysis of ALP activity in MSCs was conducted at 4, 7, and 14 days of co-culture. **(G)** The concentration of OCN in cell culture supernatant was differentially analyzed at 4, 7, and 14 days, with the supernatant collected each time to ensure that OCN accumulated for 2 days. MSCs were cultured alone without using materials as Control group, and co-cultured without using materials as Control + Mφ group. The results are presented as mean ± SD and comparised with two-way ANOVA and *post hoc* multiple comparisons through LSD or Tamhane’s T2 test, with *p < 0.05, **p < 0.01, ***p < 0.001, ****p < 0.0001 denoting significance levels, n = 3.

**FIGURE 9:**
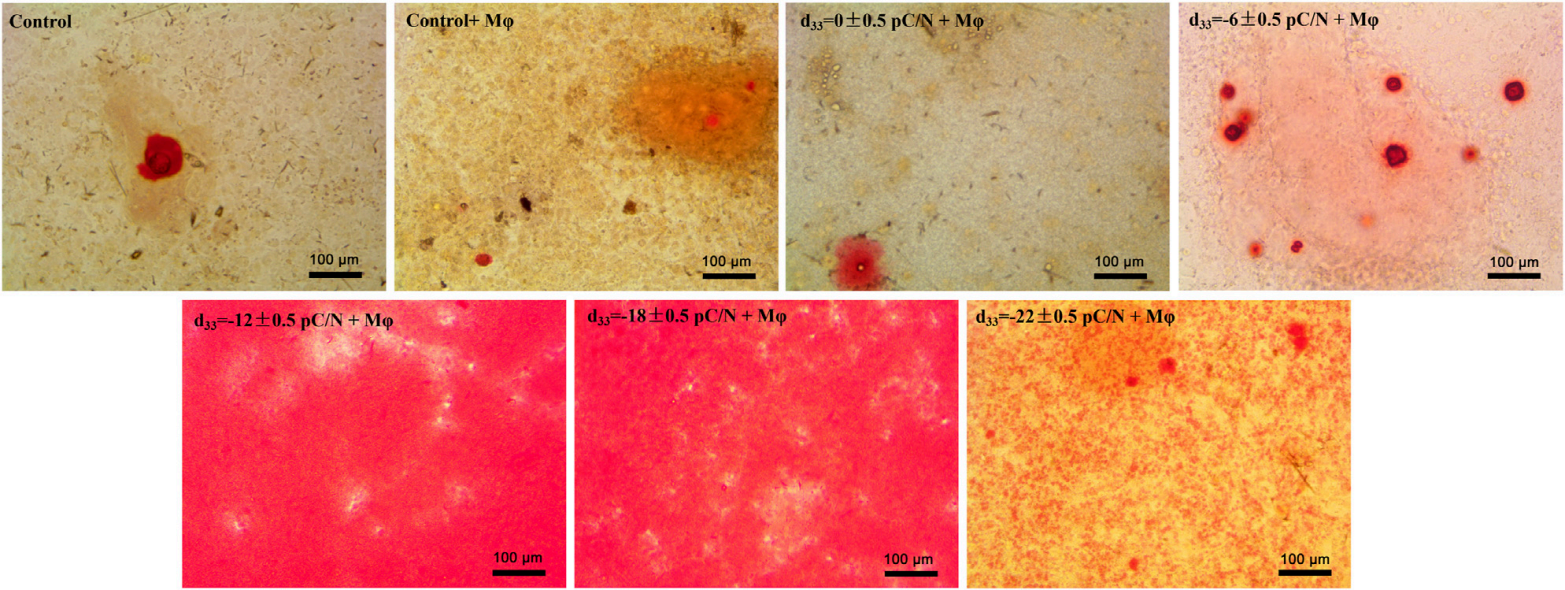
Extracellular matrix mineralization was observed on MSCs co-cultured with RAW264.7 macrophages on various surface-charged P(VDF-TrFE)/ITO planar microelectrodes over a period of 14 days. MSCs were cultured alone without using materials as Control group, and co-cultured without using materials as Control + Mφ group, n = 3.

Observing the variance in osteogenic gene expression (*Sp7*, *Runx2*, *Alpl*, *Col1a1* and *Bglap*) of MSCs at 7 and 14 days of co-culture, it was evident that the co-culture group exhibited higher expression compared to the Control group. Furthermore, the presence of surface charge within the co-culture group stimulated osteogenic gene expression. A parabolic trend was noted with an increase in the number of negative surface charge, and notably, the d_33_ = −18 ± 0.5 pC/N + Mφ group demonstrated the most profound osteogenic induction effect on MSCs (Figures 8A–E). During the co-culturing process, the activity of ALP in cells and the concentration of OCN in the cell culture supernatant increased over time (Figures 8F, G). Specifically, at 7 days, the concentration of OCN in the supernatant was significantly higher only in the d_33_ = −18 ± 0.5 pC/N + Mφ group compared to the other groups. The ALP activity at 7 and 14 days and the OCN concentration at 14 days began to show more prominent differences, with the d_33_ = −18 ± 0.5 pC/N + Mφ group consistently maintaining the highest, while the d_33_ = −22 ± 0.5 pC/N + Mφ group consistently remained lower than the d_33_ = −12 ± 0.5 pC/N + Mφ group at the 14th day. Alizarin red staining was conducted after a 14-day culture period (Figure 9). The Control, Control + Mφ, and d_33_ = 0 ± 0.5 pC/N + Mφ groups exhibited only sporadic scattered massive or spherical calcium nodules. In contrast, the d_33_ = −6 ± 0.5 pC/N + Mφ group displayed a large number of scattered calcium nodules, while the d_33_ = −22 ± 0.5 pC/N + Mφ group had its entire field covered by scattered calcium nodules. The d_33_ = −12 ± 0.5 pC/N + Mφ and d_33_ = −18 ± 0.5 pC/N + Mφ groups featured a complete occupation of the field of view by large sheets of calcium nodules.

## 4 Discussion

Upon etching the ITO planar microelectrodes, surfaces with staggered heights and regional variations in physical and chemical properties are formed (Figures 1F, I). The inconsistency of these surface properties poses challenges for studying the effects of surface potential on cells alone. To address this, a layer of P(VDF-TrFE) film is applied to the surface of the ITO planar microelectrode. This serves to alleviate the rigidity of the surface of ITO planar microelectrode (Figure 1H), resulting in a more homogeneous composition (Figure 1G), surface height (Supplementary Figure S2B), and hydrophilicity (Supplementary Figure S2C) of the material surface. Simultaneously, it increases the roughness of the material surface (Figure 1E). Moreover, studies (Naji et al., 2016) have shown that the injection of ITO nanoparticles into the peritoneum of mice induces peritonitis through the aggregation of inflammatory cells and apoptosis. Therefore, the P(VDF-TrFE) film coverage significantly reduces the release of ITO nanoparticles and enhances the overall biocompatibility of the material.

P(VDF-TrFE) thin film is an electroactive material derived from PVDF, exhibiting excellent ferroelectric and piezoelectric properties, along with superior biocompatibility (Nunes-Pereira et al., 2015; Kao et al., 2017). PVDF (Zhu and Wang, 2012) can crystallize into four different phases—alpha (α), beta (β), gamma (γ), and delta (δ)—depending on the processing conditions. The β-phase, characterized by its all-trans (TTTT) configuration, aligns molecular dipoles perpendicular to the chain axis, resulting in the highest dipole moment and superior electroactivity among the phases. Therefore, the electrical properties of the material can be assessed by the percentage of the β-phase in the film. The method described herein significantly increases the β-phase crystallinity in P(VDF-TrFE) thin films (Figures 1C, D), with a β-phase content of up to 81.76% (Figure 1C), demonstrating outstanding ferroelectric and piezoelectric characteristics.

Analysis of osteogenic genes, protein expression, and mineralization within 14 days of osteogenic induction in MSCs confirmed a parabolic trend in promoting osteogenic induction with increasing surface charge. The most robust effect on osteogenic induction in MSCs was observed with the surface charge d_33_ = −18 ± 0.5 pC/N (Figures 2, 3). Studies have shown (Jia et al., 2019) that alterations of the active site of fibronectin (Fn) adsorbed proteins can influence integrin conformation, thereby affecting the mediated Integrin/FAK/ERK signaling pathway, subsequently impacting MSCs bone formation and osteoinduction. In another study exploring the effect of persistent surface charge of P(VDF-TrFE) films on osteoinduction in MC3T3-E1 osteoblasts (Tang et al., 2018), a similar parabolic trend was observed in the level of osteogenic differentiation with increasing surface charge, indicating that excessive surface charge inhibited osteogenesis. The study also found that the binding of the Arginine-Glycine-Aspartic acid (RGD) active site of Fn to integrin β1 and the Pro-His-Ser-Arg-Asn (PHSRN) active site of Fn to integrin α5 played a significant role. Optimal facilitation of osteogenesis was observed when both active sites were perfectly bound, with the distance between them being important for Fn binding to integrin α5β1, with a distance of 35 Å being the optimal distance (Ochsenhirt et al., 2006). The d_33_ = −18 ± 0.5 pC/N surface charge in our study groups may promote the osteogenic differentiation of cells through the FAK/ERK signaling pathway by enabling the Fn active site to be fully exposed to the medium and shortening the distance between the RGD and the PHSRN for optimal integrin binding, thus facilitating osteogenesis. In subsequent studies, we plan to use HFN7.1 and mAb1937 monoclonal antibodies to respectively assess the exposure levels of the RGD and PHSRN active sites and evaluate the expression levels of proteins related to the FAK/ERK signaling pathway to investigate the regulatory mechanisms of different surface charge on MSCs at the molecular level.

Studies using the CCK-8 assay revealed that the P(VDF-TrFE)/ITO planar microelectrodes significantly impact the viability of BMSCs (Figure 2A). We believe this outcome arises because the proliferative capacity of BMSCs notably decreases following the initiation of osteogenic induction, thereby amplifying the cytotoxic effects of the material. We considered the potential of utilizing surface charge effects on other cells to indirectly promote osteogenic differentiation in BMSCs, thereby minimizing material-induced harm. Macrophages, which play a crucial and significant role in the osseointegration process and possess robust proliferative abilities, were incorporated into our analysis. Observations of macrophage viability over 7 days using the CCK-8 assay indicated minimal material toxicity impact, laying a solid cellular foundation for indirectly facilitating osteogenic differentiation in BMSCs (Figure 4A).

The impact of various surface charge on macrophage polarization can be effectively visualized through SEM (Figure 5). Macrophages adhered to P(VDF-TrFE)/ITO planar microelectrodes lacking surface charge exhibited near-spherical morphology, representing the unpolarized M0 type. However, when the planar microelectrodes carried charge, the prevalence of spherical macrophages decreased while the number of spread flattened macrophages notably increased. Western blot analysis (Figures 4B, C) confirmed that the negative surface charge of P(VDF-TrFE)/ITO planar microelectrodes predominantly induced macrophage polarization toward the M1 pro-inflammatory phenotype, with the d_33_ = −18 ± 0.5 pC/N charge presenting the strongest pro-inflammatory polarization. While the d_33_ = −22 ± 0.5 pC/N charge also promoted M1 polarization, it demonstrated a greater propensity for promoting macrophage M2 polarization.

The examination of gene expression persistence (Figure 6) indicated that the process of polarization commenced upon cell attachment, and the quantity of polarized cells elevated with the duration of culture. The d_33_ = −18 ± 0.5 pC/N group exhibited the most prominent pro-M1 polarization at the advanced stage, while the release of pro-inflammatory cytokines reached its peak at the initial stage. We attribute this occurrence to the emergence and operation of negative feedback regulation of pro-inflammatory signals by the macrophages themselves during the late stage of culture to suppress the pro-inflammatory state.

It was demonstrated (Zaveri et al., 2014) that the M1-inducing effect of surface charge on macrophage polarization is mediated through the PI3K/AKT pathway, facilitated by the binding of the RGD and PHSRN active sites of Fn to the integrin α5β1, and the P1/P2 active site of fibrinogen (Fg) to the αMβ2 integrin. A suitable surface potential can enhance Ca^2+^ inward flow by modulating voltage-gated Ca^2+^ channels, thus activating the calpain-2. The coupling of calpain-2 and talin-1 can activate the intracellular structural domain of the integrin β1 subunit, initiating the integrin-mediated TGF-β1/Smad signaling pathway, and thereby promoting macrophage M2 polarization (Ribeiro et al., 2012; Li et al., 2019). We think the d_33_ = −18 ± 0.5 pC/N surface charge have the most significant impact on the adsorbed protein conformation, resulting in optimal exposure of the RGD active site of Fn and the P1/P2 active site of Fg, facilitating integrin binding and hence exhibiting the strongest induction of macrophage M1 polarization via the PI3K/AKT pathway. The negative surface charge of the material maintains the extracellular environment in a negatively charged state, which inhibits the inward flow of Ca^2+^, diminishes the binding of calpain-2 to talin-1, and inhibits the intracellular structural domain-mediated TGF-β1/Smad signaling pathway of the integrin β1 subunit, consequently hindering macrophage M2 polarization. In molecular-level studies of macrophage regulation via surface charge, in addition to previously mentioned detection of Fn active site exposure, we will utilize anti-γ-chain monoclonal antibodies to measure the exposure of P1/P2 active sites. We will also observe intracellular and extracellular Ca^2+^ flux using Ca^2+^ fluorescent probes and assess the expression of related signaling pathway proteins.

The debate centers around the ability of M1 macrophages to promote the osteogenic differentiation of MSCs (Gong et al., 2016; Lu et al., 2017), while the promoting effect of M2 macrophages on the osteogenic differentiation of MSCs (Gong et al., 2016) and the polarization of MSCs towards M2 macrophages (Cho et al., 2014; Chen et al., 2018; Zheng et al., 2018) has been extensively validated.

In the investigation of the influence of mesenchymal stem cells (MSCs) and surface charge on macrophage polarization (Figure 7), we observed that the co-culture of MSCs and macrophages alone could directly induce the polarization of macrophages from M0 to M2-type. Furthermore, MSCs were found to mitigate the M1 polarization-promoting effect of negative surface charge on macrophages and to induce macrophage M2 polarization. Moreover, we noted that the M2 polarization-promoting effect was strengthened with an increase in culture time. Specifically, MSCs primarily stimulated the production of M2a-type macrophages whose surface marker is *Mrc1* (Figure 7A, a), and only macrophages in the d_33_ = −22 ± 0.5 pC/N + BMSCs group exhibited partial transformation into M2c-type whose surface marker is *Cd163* (Figure 7B, b) after 4 days of indirect co-culture. Notably, M2c-type macrophages demonstrated potent anti-inflammatory activity against apoptotic cells (Lurier et al., 2017). We speculate that the production of M2c-type macrophages in the d_33_ = −22 ± 0.5 pC/N + BMSCs group is primarily attributable to the inhibitory effect of planar microelectrodes with d_33_ = −22 ± 0.5 pC/N on cell viability.

In the investigation of the impact of macrophages affected by surface charge on the osteogenic differentiation of MSCs (Figures 8, 9), it was observed that the Control + Mφ group was capable of enhancing the osteogenic differentiation of MSCs in comparison to the Control group. This indicates that macrophages exert a promoting influence on the osteogenesis of MSCs, irrespective of their phenotype. Specifically, macrophages subjected to a sufficiently negative surface charge and co-cultured with MSCs significantly boosted the osteogenic differentiation of MSCs, with the d_33_ = −18 ± 0.5 pC/N + Mφ group exhibiting the most pronounced promotional effect. Some researchers (Gong et al., 2016) have asserted that M1-type macrophages impede osteogenesis in MSCs, while Nathan et al. (2019) have underscored the significance of the pro-inflammatory state of macrophages for bone repair over a period of 72–96 h. Notably, in this study, the d_33_ = −18 ± 0.5 pC/N surface charge had a more profound impact on macrophage M1 polarization in comparison to the d_33_ = −22 ± 0.5 pC/N surface charge. Additionally, M2 polarization was considerably weaker in the d_33_ = −18 ± 0.5 pC/N co-culture group than in the d_33_ = −22 ± 0.5 pC/N co-culture group during the co-culturing process. However, despite this, the d_33_ = −18 ± 0.5 pC/N co-culture group exhibited a greater promotion of MSCs osteogenesis than the d_33_ = −22 ± 0.5 pC/N co-culture group. This confirms that a consistently high-intensity macrophage M2 polarization phenotype does not optimize the macrophage-induced osteogenesis of MSCs, underscoring the necessity and importance of an early macrophage M1 pro-inflammatory phenotype for the osteogenic effect on MSCs.

## 5 Conclusion

The negatively charged surface with a piezoelectric coefficient of −18 has a significant promoting effect on both the M1-type polarization of macrophages and the osteogenic differentiation of MSCs. Moreover, macrophages under its regulation exhibit the strongest induction of osteogenic differentiation in MSCs. The promotion of M2 polarization of macrophages by MSCs competes and prevails over the promotion of M1 polarization of macrophages induced by the negative surface charge. Furthermore, the osteogenic promotion of MSCs by macrophages, which are subjected to the superimposed promotion of M2 polarization of both MSCs and a high negative surface charge (d_33_ = −22 ± 0.5 pC/N), is significantly lower than the osteogenic promotion by macrophages, which are subjected to the competitive induction of strong M1 polarization by an appropriate surface charge and the M2 polarization by MSCs. This confirms the necessity and importance of the macrophage M1 pro-inflammatory phenotype for the osteogenic effect of MSCs.

## Data Availability

The raw data supporting the conclusion of this article will be made available by the authors, without undue reservation.
