# Supplementary material for: Investigation of the impact of planar microelectrodes on macrophage-mediated mesenchymal stem cell osteogenesis
Source: Front Cell Dev Biol. 2024 Jun 3;12:1401917. doi: 10.3389/fcell.2024.1401917 (PMC11180747; doi:10.3389/fcell.2024.1401917)
Supplement: Supplementary file 1 [file DataSheet1.docx]

Supplementary Material


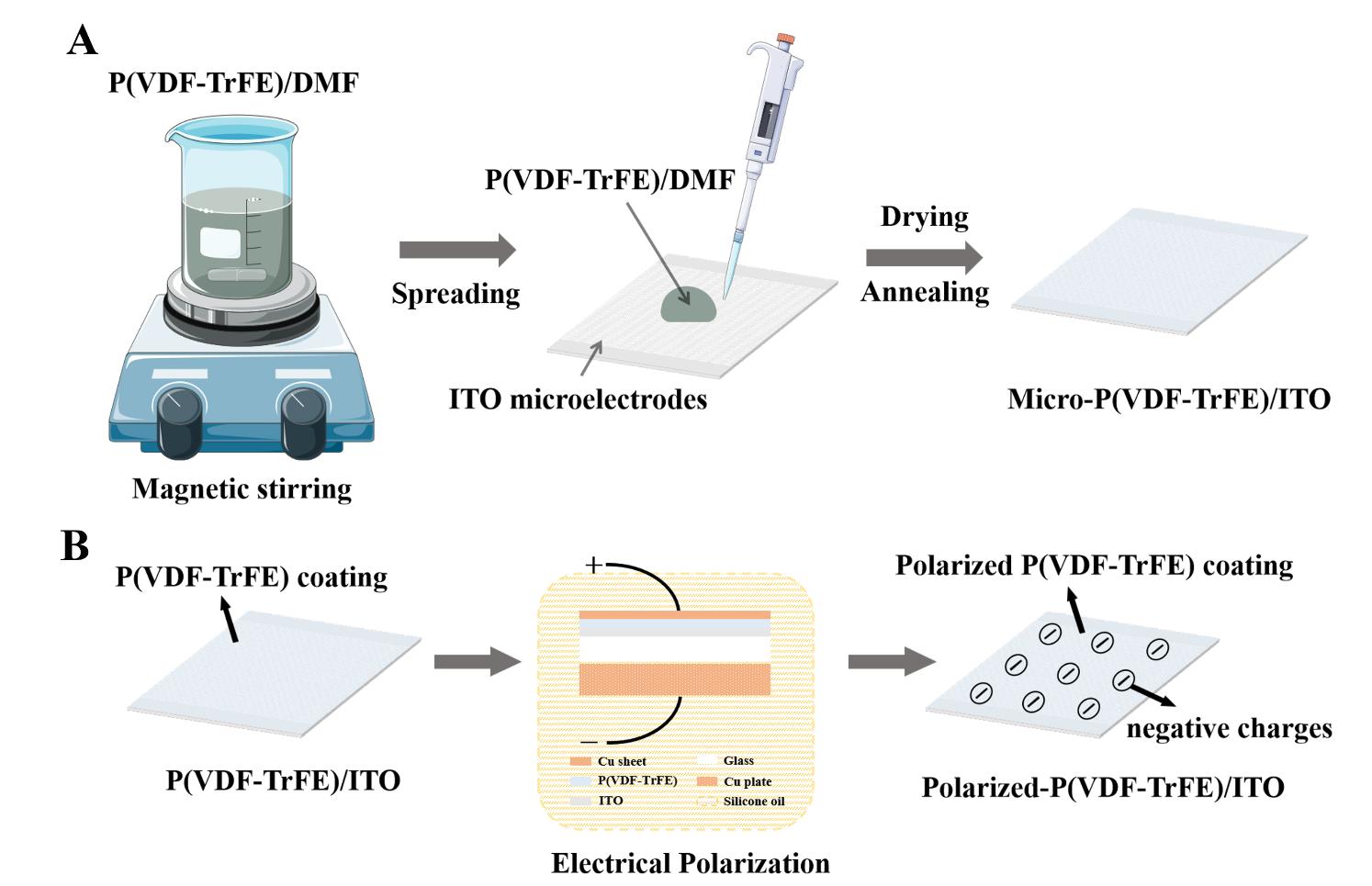
Supplementary Figure 1 Preparation and polarization methods of P(VDF-TrFE)/ITO planar microelectrodes. (A) Preparation method of P (VDF-TrFE)/ITO planar microelectrodes. (B) Using direct contact polarization to carry different surface charges on P (VDF-TrFE)/ITO planar microelectrodes.


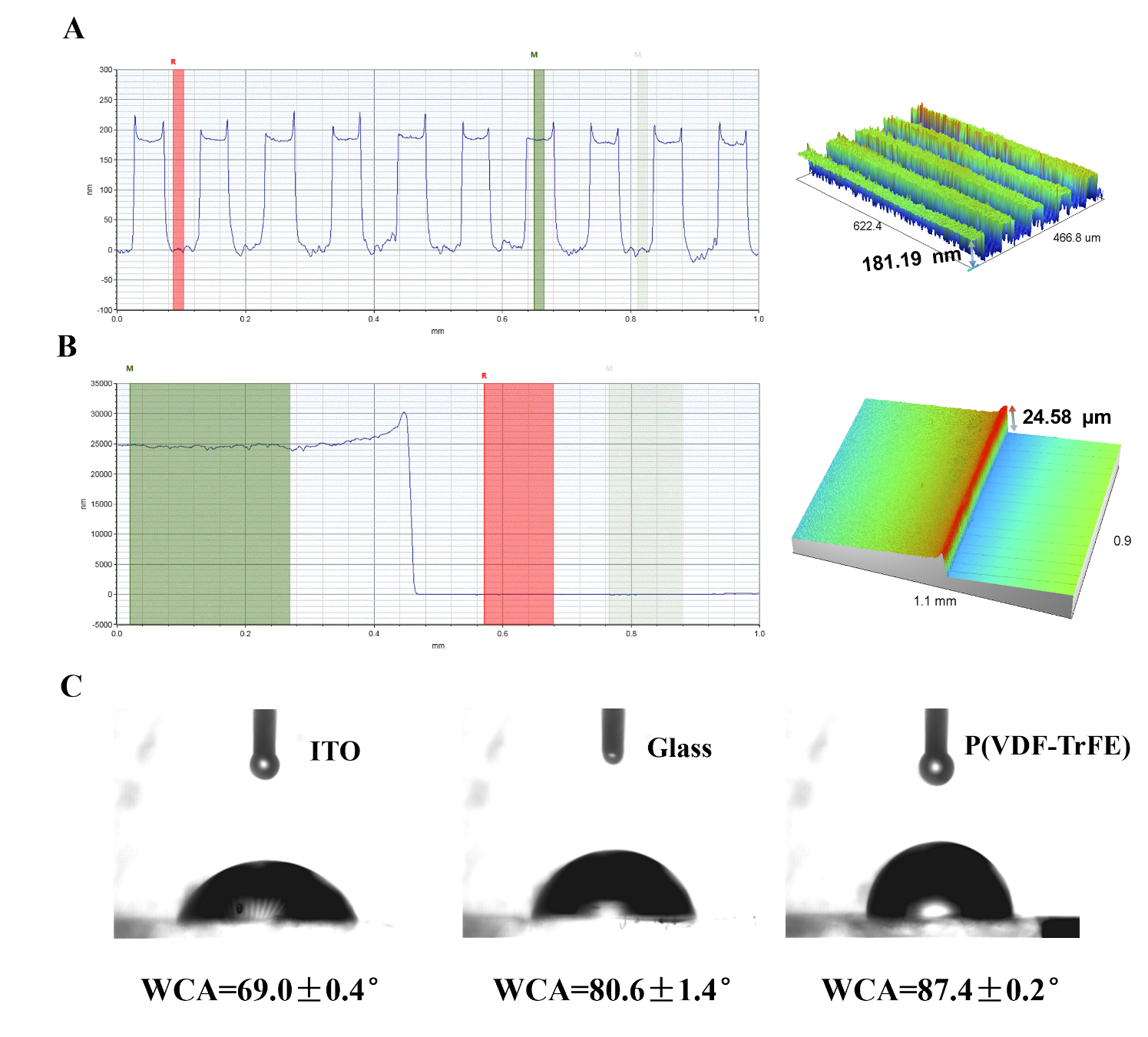


**Supplementary Figure 2** The height difference and hydrophobicity of P (VDF-TrFE)/ITO planar microelectrodes. (A) Measuring ITO planar microelectrode height difference by step profiler (DEKTAK-XT). (B) Measuring the thickness of P (VDF-TrFE) thin film by step profiler. The probe crossed the surface thin film between the electrode region and the etching region. (C) Characterization of material surface hydrophobicity using static contact angle (WAC) of ITO area, glass area and P(VDF-TrFE) thin film.
